# The earliest diverging extant scleractinian corals recovered by mitochondrial genomes

**DOI:** 10.1038/s41598-020-77763-y

**Published:** 2020-11-26

**Authors:** Isabela G. L. Seiblitz, Kátia C. C. Capel, Jarosław Stolarski, Zheng Bin Randolph Quek, Danwei Huang, Marcelo V. Kitahara

**Affiliations:** 1 Departamento de Ciências do Mar, Universidade Federal de São Paulo, Santos, São Paulo Brazil; 2 Centro de Biologia Marinha, Universidade de São Paulo, São Sebastião, São Paulo Brazil; 3 Institute of Paleobiology, Polish Academy of Sciences, Warsaw, Poland; 4 Department of Biological Sciences, National University of Singapore, Singapore, Singapore; 5 Tropical Marine Science Institute, National University of Singapore, Singapore, Singapore

**Keywords:** Phylogenomics, Evolution

## Abstract

Evolutionary reconstructions of scleractinian corals have a discrepant proportion of zooxanthellate reef-building species in relation to their azooxanthellate deep-sea counterparts. In particular, the earliest diverging “Basal” lineage remains poorly studied compared to “Robust” and “Complex” corals. The lack of data from corals other than reef-building species impairs a broader understanding of scleractinian evolution. Here, based on complete mitogenomes, the early onset of azooxanthellate corals is explored focusing on one of the most morphologically distinct families, Micrabaciidae. Sequenced on both Illumina and Sanger platforms, mitogenomes of four micrabaciids range from 19,048 to 19,542 bp and have gene content and order similar to the majority of scleractinians. Phylogenies containing all mitochondrial genes confirm the monophyly of Micrabaciidae as a sister group to the rest of Scleractinia. This topology not only corroborates the hypothesis of a solitary and azooxanthellate ancestor for the order, but also agrees with the unique skeletal microstructure previously found in the family. Moreover, the early-diverging position of micrabaciids followed by gardineriids reinforces the previously observed macromorphological similarities between micrabaciids and Corallimorpharia as well as its microstructural differences with Gardineriidae. The fact that both families share features with family Kilbuchophylliidae ultimately points towards a Middle Ordovician origin for Scleractinia.

## Introduction

Scleractinian corals are renowned for their capacity to create spectacular shallow-water calcium carbonate reef structures. Azooxanthellate scleractinians—corals that do not establish a symbiotic relationship with dinoflagellates of the family Symbiodiniaceae^1,2^—total approximately the same number of extant zooxanthellate species of the order, but are not restricted to tropical, shallow-waters as the latter. The early evolutionary history of the order Scleractinia has been the subject of intense scientific debate. For example, the monophyly of this order has been challenged since the order Corallimorpharia was previously recovered as a clade nested within the main scleractinian lineages (“naked coral” hypothesis^3^). Nevertheless, it has been shown that such a corallimorpharian position was an artefact of the use of amino acid sequences in phylogenetic analyses^4^. Furthermore, the evolutionary history of the main reef builders has also attracted great attention as the “molecular revolution” challenged the long-established morphological systematics especially for the higher taxonomic ranks^5–11^. Originally, based on gross morphology, the order Scleractinia was divided into five^12^, or thirteen^13^ suborders. However, more recently, molecular data pointed to three main clades: “Basal”; “Complex”; and “Robust” corals^4,11,14–16^.

**Figure 1:**
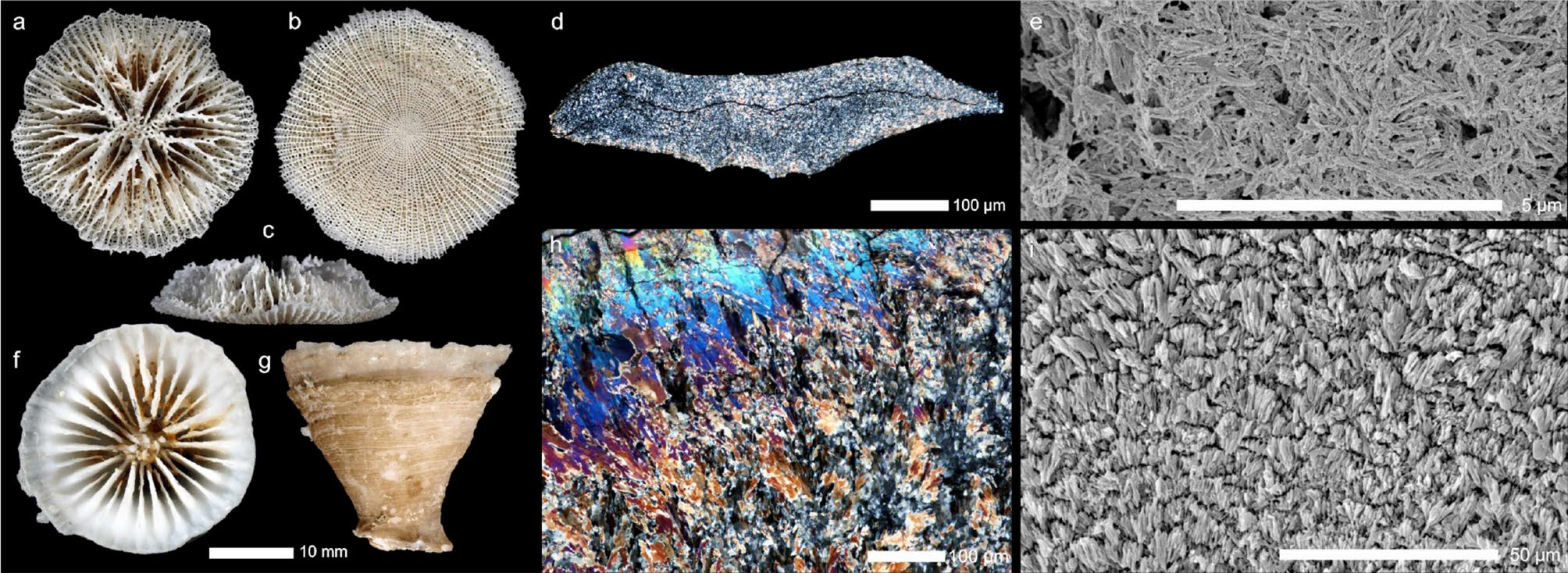
Skeletal morphology and microstructure of representatives of the basal scleractinian clade. While micrabaciids typically have a light, lace-like skeleton with perforated walls and septa (**a**–**c**, *Letepsammia formosissima* (Moseley, 1876) in distal, basal and lateral views, respectively), gardineriids have very robust coralla (**f**–**g**, *Gardineria hawaiiensis* Vaughan, 1907) in distal and lateral views, respectively). In micrabaciids (here *L. formosissima*) Thickening Deposits (TDs) are composed of an irregular meshwork of fiber bundles oriented sub-parallel to the skeleton surface (**d**,**e**), whereas in gardineriids (*G. hawaiiensis*) TDs are arranged in small bundles of fibers oriented approximately perpendicular to the skeleton surface. Consequently, micrabaciid TDs show variable crystallographic orientation (**d**, seen as lack of larger areas of similar vivid interference colors in polarized light), whereas in gardineriids TDs are crystallographically ordered and larger areas of similar vivid interference colors are visible in polarized light (**h**). Thin-sections in polarized microscope views (**d**,**h**), and polished and lightly etched sections in Scanning Electron Microscopy views (**e**,**i**).

In contrast to widely accepted Triassic emergence of Scleractinia, divergence of the earliest scleractinian clade with extant representatives (families Micrabaciidae and Gardineriidae) was suggested to have occurred between the Ordovician and Silurian, around 425 million years ago (mya)^14^, or in the Silurian, 407 mya^16^. Although recovering a slightly later onset, a recent study by Quattrini and collaborators^17^ has also pointed to a Paleozoic origin for the order. Composed of exclusively azooxanthellate taxa^18^, representatives of Micrabaciidae share some morphological skeletal characters (septal bifurcations) with the Ordovician Kilbuchophylliidae (~ 460 mya), but otherwise represent a morphologically unique coral group^19^. On the other hand, gardineriids develop usually a thick, exclusively epithecal wall^20^ typical of some of the oldest known solitary Mesozoic (Middle Triassic) corals^14^ (~ 230 mya; Fig. 1a–c,f,g). Gardineriidae is also composed exclusively of azooxanthellate solitary corals, occurring from 2 to 1200 m depth^21^. Due to the unique micrabaciid microstructure^14,19,22,23^, which is not comparable to that of gardineriids (Fig. 1d,e,h,i) nor any other modern or fossil scleractinian coral, the phylogenetic position of micrabaciids within the “Basal” clade is intriguing. Known to occur from 15 m to as deep as 5000 m^21,24^, representatives of the Micrabaciidae are characterized by having free-living, solitary polyps with their porous skeleton completely enwrapped by soft tissue^25^. The oldest known micrabaciid fossil dates from shallow-water Mesozoic strata. However, with their skeletons enwrapped by tissue, like extant, shallow-water fungiids and turbinoliids^26–28^, it is possible that they are capable of automobility, an ability also documented in deep-water *Flabellum*^29^ and discussed to be present in Paleozoic corals, such as *Palaeacis regularis* from the Permian^30^. Furthermore, such ability together with no skeletal exposure to the environment, most likely favoured these organisms to occur in deepwater^24^ and to withstand the calcium-limited conditions^31^ at depths below the aragonite saturation horizon^32^. At the same time, calcification at great depths is physiologically demanding thus a trend towards skeleton lightening (increased porosity) is also observed in micrabaciid evolution.

**Figure 2:**
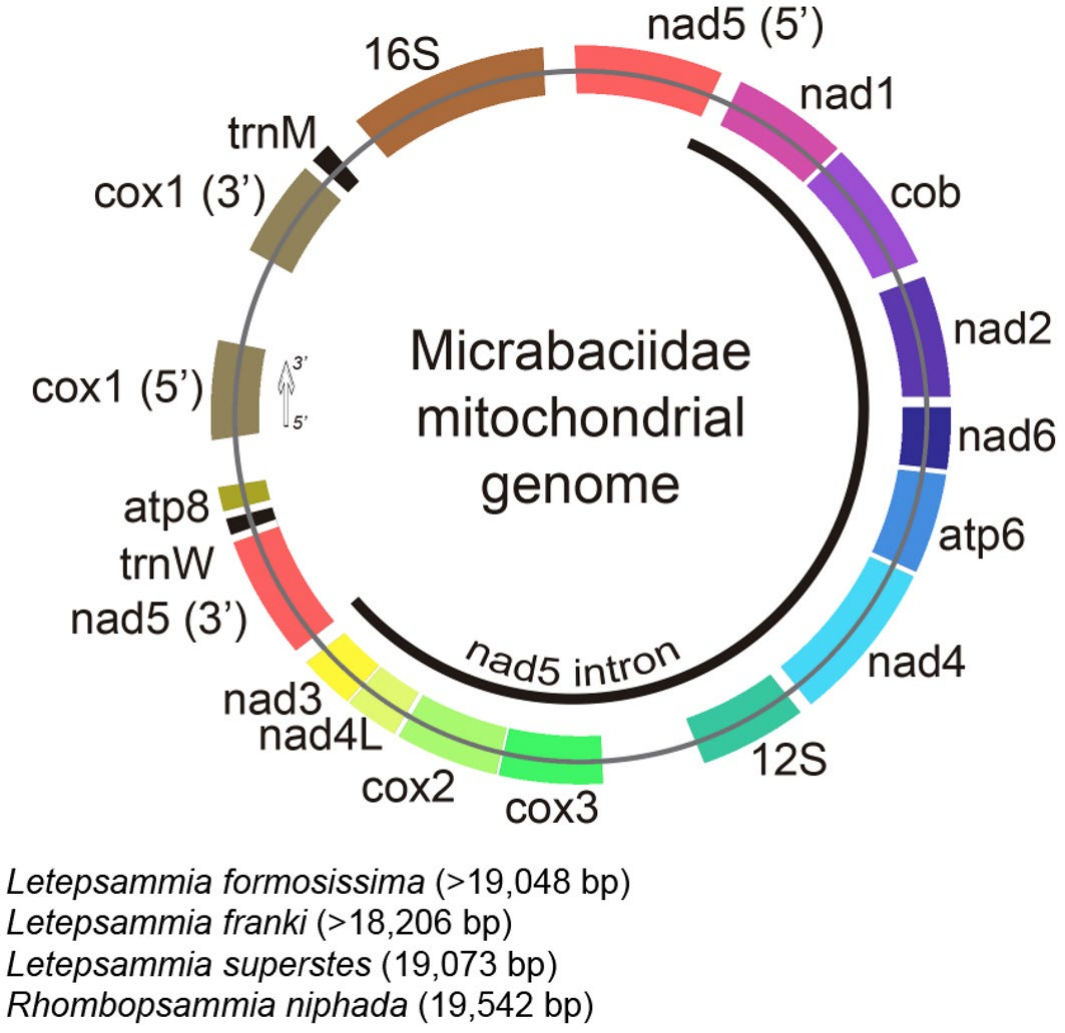
Gene content, order and sizes of Micrabaciidae mitogenomes. 5′ and 3′ indicate transcription direction and gene abbreviations are similar to those used in the text. For *L. franki* and *L. formosissima*, sizes are based on data obtained in five contigs and one incomplete contig, respectively.

Overall, scleractinian mitogenomes have unique features, such as few transfer RNA genes (trnW, which is duplicated in *Seriatopora* and *Stylophora*, and trnM^4,33,34^), as well as the occurrence of introns in two protein-coding genes: *nad5* and *cox1*. In *nad5*, this feature is present in all scleractinian mitogenomes determined to date^3,35,36^, while the *cox1* intron is absent in some species and appears to have been regained at least five times in the “Robust” coral clade^37,38^. Regarding gene order, the same pattern seems to be shared among the majority of species sequenced to date^35^, except for *Madrepora* spp.^35,39^, *Desmophyllum dianthus* and *D. pertusum*^35,36,40,41^, and *Solenosmilia variabilis* (Fig. 2).

To date, the majority of data used to investigate the evolutionary history of scleractinian corals is derived from shallow-water zooxanthellate species, limiting a broader understanding of several aspects of the evolution of the order^11,15^. Such a pattern has been reproduced for genomic and transcriptomic data. A search for scleractinian mitogenomes on Nucleotide database^42^ and genomic or transcriptomic data on Sequence Read Archive^43^ (excluding metagenomic data and both accessed on May, 2020) turned up 81 mitogenomes and 107 nuclear genomic/transcriptomic-level datasets from colonial and zooxanthellate species compared to 11 and 24, respectively, from solitary or azooxanthellate/facultative species. Among azooxanthellate species, only two mitogenomes (*Fungiacyathus stephanus* [JF825138] and *Gardineria hawaiiensis* [MT376619]) and seven nuclear (*Balanophyllia elegans*, *Balanophyllia europaea*, *Caryophyllia arnoldi*, *Flabellum alabastrum*, *Paraconotrochus antarcticus*, *Rhizotrochus* sp., *Thecopsammia* sp.) datasets were from solitary species. Also, apart from the mitogenomes presented herein, the only available data (mitochondrial and nuclear genomes or transcriptomes) from "Basal" representatives is resumed to the mitogenome of *Gardineria hawaiiensis*^4^ (Gardineriidae). Therefore, in this study, we refined the understanding of the evolutionary history of these early diverging lineages, more specifically by determining the mitogenomes of four micrabaciids (*Letepsammia franki*, *L. superstes*, *L. formosissima* and *Rhombopsammia niphada*). Together, the results presented here shed light on the Early Paleozoic origin of the order but also raise further questions on the discrepancy between mitochondrial and nuclear-based phylogenies within Scleractinia, a phenomenon detected for Cnidaria^44^ and other animal groups^45,46^.

## Results

Generated raw reads ranged from 3,037,202 to 5,605,634 (MiSeq run) and corresponded to 26,584,520 and 29,910,418 (NextSeq run). Trimmed reads ranged from 2,767,107 to 4,840,710 and 14,566,182 to 15,824,076, respectively (Supplementary Table S1 online). Mitogenomes determined herein (Table 1) were each assembled in one contig, all above 19 kbp (*Rhombopsammia niphada*: 19,542 bp; *Letepsammia formosissima*: 19,048 bp; *Letepsammia superstes*: 19,073 bp; see number of reads mapped in each assembly on Supplementary Table S1 online), although that from *L. formosissima* lacks a part of the 16S ribosomal gene (probably around ~ 30 bp; Table 2). Sanger data from *Letepsammia franki* was assembled into five contigs comprising 18,206 bp in total and the only absent gene was trnW. Micrabaciidae mitogenomes have 37.8–37.9% GC levels, values slightly lower than that observed for *G. hawaiiensis* and Corallimorpharia (both with 39.7%). Nevertheless, “Complex” corals include a wide range of values, from 36.2% in *Porites lobata* to 40.5% in *Pavona clavus* (see Table 1).

**Table 1:** Lengths and GC contents of mitogenomes included in the phylogeny.

Species	Accession	Length (bp)	GC content (%)
**Actiniaria**			
*Metridium senile*	AF000023	17,443	38.1
*Nematostella *sp.	DQ643835	16,389	39.1
**Corallimorpharia**			
*Corallimorphus profundus*	KP938440	20,488	39.7
*Discosoma nummiforme*	KP938434	20,925	39.0
*Pseudocorynactis *sp.	KP938437	21,239	39.1
**Micrabaciidae**^**a**^			
*Letepsammia formosissima*^b^	MT705247	19,048	37.9
*Letepsammia franki*^b^	MT706036–MT706040	18,206	37.8
*Letepsammia superstes*	MT706035	19,073	37.9
*Rhombopsammia niphada*	MT706034	19,542	37.8
**Gardineriidae**			
*Gardineria hawaiiensis*	MT376619	19,429	39.7
**“Complex”**			
*Acropora tenuis*	AF338425	18,338	38.0
*Agaricia humilis*	DQ643831	18,735	40.4
*Alveopora* sp.	KJ634271	18,146	37.9
*Anacropora matthai*	AY903295	17,888	38.4
*Dendrophyllia arbuscula*	KR824937	19,069	37.3
*Fimbriaphyllia ancora*	JF825139	18,875	37.8
*Fungiacyathus stephanus*	JF825138	19,381	37.8
*Galaxea fascicularis*	KU159433	18,751	38.3
*Goniopora columna*	JF825141	18,766	37.1
*Pavona clavus*	DQ643836	18,315	40.5
*Porites lobata*	KU572435	18,647	36.2
*Porites porites*	DQ643837	18,648	36.3
*Pseudosiderastrea tayamai*	KP260633	19,475	36.3
*Tubastraea coccinea*	KX024566	19,094	37.2
*Turbinaria peltata*	KJ725201	18,966	37.0
**“Robust”**			
*Astrangia* sp.	DQ643832	14,853	31.9
*Colpophyllia natans*	DQ643833	16,906	33.6
*Cyphastrea serailia*	KY094484	17,138	33.5
*Desmophyllum dianthus*	KX000893	16,310	35.1
*Desmophyllum pertusum*	KC875348	16,149	34.9
*Dipsastraea rotumana*	KY094481	16,466	33.2
*Echinophyllia aspera*	MG792550	17,697	34.1
*Favites abdita*	KY094479	17,825	33.8
*Hydnophora exesa*	KY094486	17,790	33.4
*Madracis decactis*^b^	KX982259	16,970	31.7
*Madracis mirabilis*	EU400212	16,951	31.7
*Madrepora oculata*	JX236041	15,841	30.3
*Mussa angulosa*	DQ643834	17,245	33.7
*Orbicella annularis*^b^	AP008974	16,138	33.6
*Platygyra carnosa*	JX911333	16,463	33.0
*Plesiastrea versipora*	MH025639	15,320	32.0
*Pocillopora damicornis*	EU400213	17,425	30.2
*Pocillopora eydouxi*	EF526303	17,422	30.1
*Polycyathus* sp.	JF825140	15,357	29.1
*Sclerophyllia maxima*	FO904931	18,168	33.7
*Solenosmilia variabilis*	KM609293	15,968	34.7

^a^Samples sequenced in this study.

^b^Mitogenomes that were not circularized successfully or were stated as linear or incomplete on NCBI.

**Table 2:** Micrabaciidae mitochondrial gene content.

Gene/IGR/intron	*Letepsammia formosissima*			*Letepsammia superstes*			*Rhombopsammia niphada*			*Letepsammia franki*		
	Position	Codons^b^	Length	Position	Codons^b^	Length	Position	Codons^b^	Length	Position^a^	Codons^b^	Length
nad5-5′	342–1061	GTG/GGT	720	1–720	GTG/GGT	720	1–720	GTG/GGT	720	(A) 1310–2029	GTG/GGT	720
igr1	–	–	247	–	–	247	–	–	247	–	–	247
nad1	1309–2292	ATG/TAA	984	968–1951	ATG/TAA	984	968–1951	ATG/TAA	984	(A) 2277–3065	ATG/TAA	789
igr2	–	–	57	–	–	57	–	–	57	–	–	N.A.
coxb	2350–3522	ATG/TAA	1173	2009–3181	ATG/TAA	1173	2009–3181	ATG/TAA	1173	(B) 1–540	TCC^c^/TAA	540
igr3	–	–	336	–	–	336	–	–	336	–	–	336
nad2	3859–4956	ATG/TAA	1098	3518–4615	ATG/TAA	1098	3518–4615	ATG/TAA	1098	(B) 877–1974	ATG/TAA	1098
igr4	–	–	88	–	–	88	–	–	89	–	–	88
nad6	5045–5605	ATG/TAA	561	4704–5264	ATG/TAA	561	4705–5265	ATG/TAA	561	(B) 2063–2623	ATG/TAA	561
igr5	–	–	16	–	–	16	–	–	16	–	–	N.A.
atp6	5622–6320	ATG/TAG	699	5281–5979	ATG/TAG	699	5282–5980	ATG/TAG	699	(C) 15–485	TCT^c^/TAG	471
igr6	–	–	45	–	–	45	–	–	45	–	–	45
nad4	6366–7841	GTG/TAG	1,476	6025–7500	GTG/TAG	1476	6026–7501	GTG/TAG	1476	(C) 531–2006	GTG/TAG	1476
igr7	–	–	188	–	–	188	–	–	176	–	–	176
12S	8030–8977	–	948	7689–8638	–	950	7678–8639	–	962	(C) 2183–3145	–	963
igr8	–	–	869	–	–	870	–	–	1338	–	–	870
cox3	9847–10,635	ATG/TAG	789	9509–10,297	ATG/TAG	789	9978–10,766	ATG/TAG	789	(C) 4016–4804	ATG/TAG	789
igr9	–	–	8	–	–	8	–	–	8	–	–	8
cox2	10,644–11,387	ATG/TAG	744	10,306–11,049	ATG/TAG	744	10,775–11,518	ATG/TAG	744	(C) 4813–5556	ATG/TAG	744
igr10	–	–	17	–	–	17	–	–	17	–	–	17
nad4L	11,405–11,704	GTG/TAA	300	11,067–11,366	GTG/TAA	300	11,536–11,835	GTG/TAA	300	(C) 5574–5873	GTG/TAA	300
igr11	–	–	15	–	–	15	–	–	15	–	–	15
nad3	11,720–12,076	GTG/TAG	357	11,382–11,738	GTG/TAG	357	11,851–12,207	GTG/TAG	357	(C) 5889–6245	GTG/TAG	357
igr12	–	–	140	–	–	140	–	–	140	–	–	140
nad5-3′	12,217–13,296	ATG/TAG	1080	11,879–12,958	ATG/TAG	1080	12,348–13,427	ATG/TAG	1080	(C) 6,386–7,465	ATG/TAG	1080
igr13	–	–	38	–	–	26	–	–	26	–	–	N.A.
trnW	13,335–13,404	TCA	70	12,985–13,054	TCA	70	13,454–13,523	TCA	70	N.A.	N.A.	N.A.
igr14	–	–	32	–	–	34	–	–	34	–	–	N.A.
atp8	13,437–13,661	ATG/TAA	225	13,089–13,313	ATG/TAA	225	13,558–13,782	ATG/TAA	225	(D) 49–273	ATG/TAA	225
igr15	–	–	765	–	–	765	–	–	765	–	–	765
cox1	14,427–16,953	ATG/TAA	2527	14,079–16,605	ATG/TA A	2527	14,548–17,074	ATG/TAA	2527	(D) 1039–1849; (E) 1–657	ATG/–; – –/TAA	811; 657 (total 1,468)
igr16	–	–	97	–	–	97	–	–	97	–	–	97
trnM	17,051–17,121	CAT	71	16,703–16,773	CAT	71	17,172–17,242	CAT	71	(E) 755–825	CAT	71
igr17	–	–	260	–	–	260	–	–	260	–	–	230
16S	1–188; 17,382–19,048	–	188; 1667	17,034–18,920	–	1887	17,503–19,389	–	1887	(E) 1056–1773; (A) 1–1156	–	718; 1156 (total 1874)
igr18	–	–	153	–	–	153	–	–	153	–	–	153
cox1 intron	15,231–16,257	–	1027	14,883–15,909	–	1027	15,352–16,378	–	1027	N.A.	N.A.	N.A.
nad5 intron	1062–12,216	–	11,155	721–11,878	–	11,158	721–12,347	–	11,627	N.A.	N.A.	N.A.

^a^Letters in parentheses indicate different *L. franki* Sanger sequencing contigs to which each gene belongs.

^b^For tRNA genes, anticodons.

^c^"Start codon" annotated by MITOS2 for an incompletely assembled gene.

**Figure 3:**
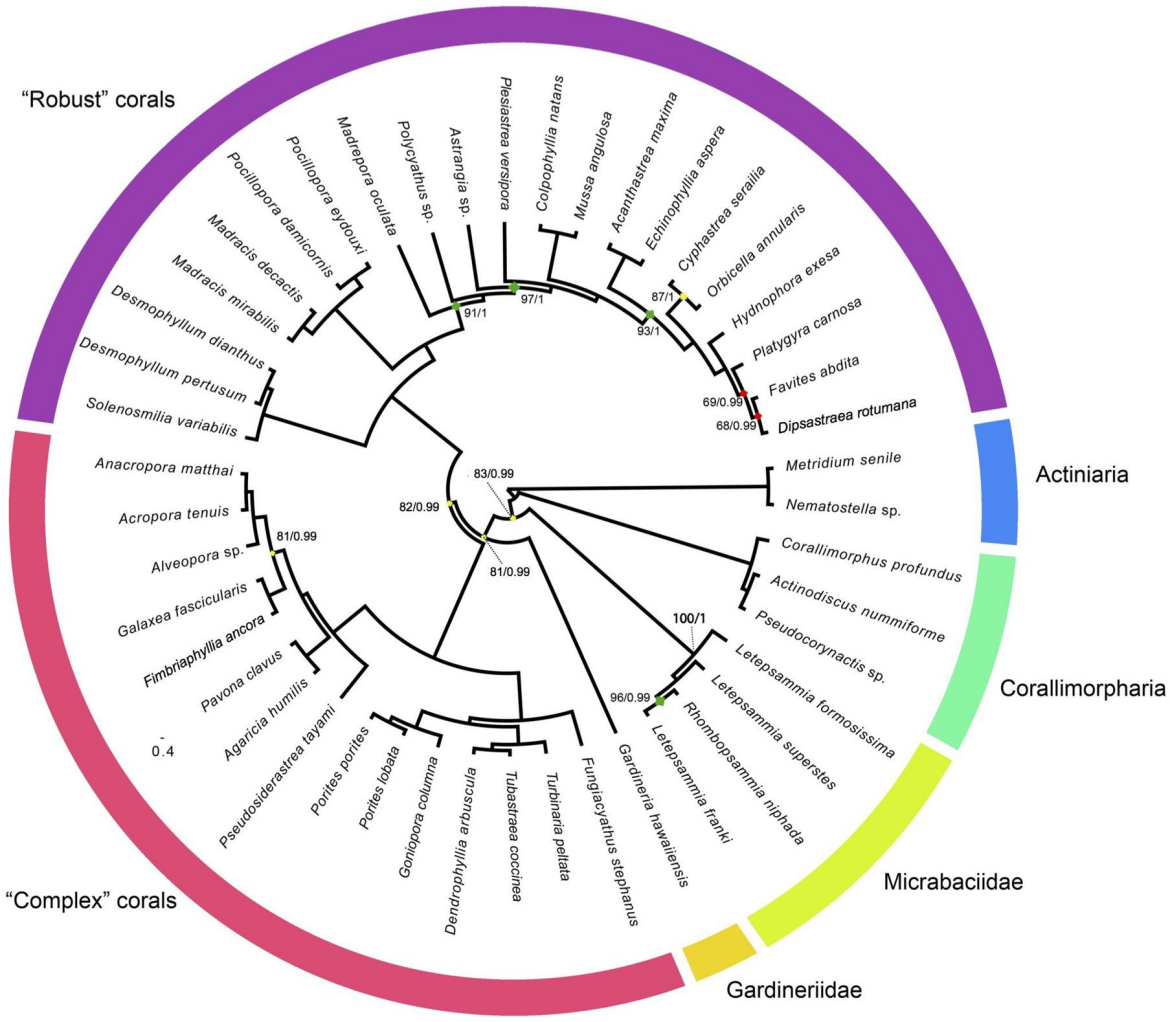
Maximum likelihood (ML) partitioned phylogeny reconstructed on RaxML using GTR model with gamma distribution and 1000 bootstrap replicates. Node support values (ML bootstrap and Bayesian posterior probabilities) are indicated only adjacent to nodes that did not display full statistical support.

Gene number and order mirrors that observed in the majority of scleractinian corals (13 protein coding genes, two ribosomal RNA genes, and two transfer RNA genes), comprising two genes coding for ATP synthase subunits (*atp6* and *atp8*), seven for NADH dehydrogenase subunits (*nad4L* and *nad1–6*), three for cytochrome c oxidase subunits (*cox1–3*), and one for cytochrome b oxidase (*coxb*). *Cox1* has a 1027 bp long intron in all three Illumina sequenced species and the intron in *nad5* includes 11 genes and is 11,627 (*R. niphada*), 11,155 (*L. formosissima*) and 11,158 (*L. superstes*) bp long (Fig. 2). Genes and intergenic regions (IGRs) are similar in length among *R. niphada*, *L. formosissima* and *L. superstes* in almost all cases, except for two genes (*nad1* and 12S) and six IGRs (igr4, igr7, igr8, igr13, igr14, igr17; Table 2)*.* Micrabaciid mitochondrial genes start codons are ATG (N = 9) and GTG (N = 4) (Table 2), while stop codons are more equitatively shared between TAA (N = 7) and TAG (N = 6). The evolutionary reconstruction using mitogenome data (Fig. 3) recovered the family Micrabaciidae as monophyletic with high statistical support (ML: 100%; BI: 1), and placed it as a sister group to all other scleractinians (i.e. Gardineriidae + “Robust” + “Complex”) with moderate to high support (ML: 83%; BI: 0.99). The family Gardineriidae was recovered as a lineage sister to “Complex” and “Robust” with moderate to high support (ML: 81%; BI: 0.99). In “Complex”, all nodes except one (Euphylliidae + Acroporidae; ML: 81%; BI: 0.99) displayed maximum values of support, while in “Robust” corals, six had non-full support values (inside family Merulinidae), being three of them with low (clade comprising *Platygyra carnosa*, *Favites abdita* and *Dipsastraea rotumana*; ML: 69% and 68%; BI: 0.99 for both) and moderate to high (*Orbicella annularis* + *Cyphastrea serailia*; ML: 87%; BI: 1) support values.

## Discussion

The phylogenetic analysis presented here indicates a paraphyly of the previously thought earliest diverging scleractinian “Basal” group^11,14–16^, represented by the two families Micrabaciidae and Gardineriidae. Based on all mitochondrial genes, the recovered phylogeny suggests that the micrabaciids were the first to diverge, being a sister group to all other scleractinians, including gardineriids. These results add further evidence for the hypothesis of solitary azooxanthellate corals as origin for the group.

Despite its crucial relevance for improving our understanding of the deep evolutionary patterns in Scleractinia, phylogenetic analyses have been based on limited data from a few partial genes (majoritarily *cox1*, 16S rDNA, 18S rDNA and 28S rDNA^14–16^). Only recently, using 933 loci (278,819 bp) captured with a targeted-enrichment approach, Quattrini et al.^17^ recovered the family Micrabaciidae as the first diverging lineage within “Robust” corals. Nevertheless, they lack representatives of Gardineriidae or other exclusively deep-water azooxanthellate families, such as Deltocyathidae and Anthemiphylliidae. On one hand, Anthozoa mitochondrial genes tend to evolve at slower rates in comparison with other metazoans^47,48^, thus favouring their application to investigate Scleractinia relationships through deep time or among taxonomic ranks above genus. On the other hand, mitogenomes may be genetically saturated, making them problematic for studying deeper levels of relationship inside Cnidaria (e.g. class level^44^). Therefore, sequencing of more mitogenomes and tests for saturation will elucidate the utility of mitochondrial genes in clarifying deep phylogenies within Scleractinia.

In general, micrabaciid mitogenomes have similar sizes to those of *G. hawaiiensis* and some “Complex” corals (e.g. *Tubastraea coccinea*, *Dendrophyllia arbuscula*, *Fungiacyathus stephanus,* and *Pseudosiderastrea tayamai*; Table 1). Among the mitogenomes sequenced herein, differences in length were mainly due to IGR sizes rather than among genes. A similar length variation pattern has previously been observed for the entire order and supports our findings for this family^35^. At 19,542 bp (Fig. 2), *R. niphada* holds the longest Scleractinia mitogenome known to date, followed by two *Pseudosiderastrea* species with 19,475 bp^49^. Micrabaciids were expected to bear long mitogenomes (> 19 kbp) based on their phylogenetic placement since the mitogenome of *Gardineria hawaiiensis* is 19,429 bp long^4^ and the overall size of scleractinian mitogenomes appears to be shrinking in relation to Corallimorpharia (between 1 and 6 kbp) and also within the order (i.e. “Robust” corals have 2–3 kbp shorter mitogenomes than “Complex” corals). Nevertheless, a clear pattern is still not evident in the class Anthozoa as a whole. While octocorals seem to present a narrower range of variation regarding mitogenome size (18 to 19.8 kbp^50,51^), hexacorals show a broader range. Actiniarian mitogenomes vary between 16 and 20 kbp, which is close to that observed in scleractinians^52,53^. Antipatharia ranges from 18 to 20 kbp^54–56^ and Zoantharia includes 20-kbp long mitogenomes, similar to Corallimorpharia^57,58^.

Different from size correlations, the recovered topology does not match previous reconstructions that included “Basal” lineages^11,14,15,59^ (Fig. 3), with a few exceptions such as reconstructions based on 28S rDNA and mitochondrial 16S rDNA that recovered Micrabaciidae and Gardineriidae as a polytomy and a paraphyly, respectively^14^. Here, micrabaciids and gardineriids were recovered in a paraphyly, the former being the earliest to diverge. Notably, Micrabaciidae shares anatomical features in common with corallimorpharians, including the presence of a thick mesoglea^60^ and acrospheres positioned around and above the polyps^61^. The molecular discrepancy between Gardineriidae and Micrabaciidae is strongly supported by differences in microstructural organization of their skeleton. For example, although the presence of an epithecal wall is common in many fossil and extant scleractinians, its occurrence as the only wall of the corallum, like in gardineriids, is exclusive in modern corals, but seems to have been a more common feature in early Mesozoic corals^20,62^. In contrast to the Gardineriidae skeletal thickening deposits (TD) (i.e. bundles of fibers arranged perpendicularly to the growing surfaces), micrabaciid TD are shaped in form of chip-like fiber bundles, sub-parallel to the skeletal surfaces, creating an irregular meshwork within the skeleton, which is not comparable with any microstructural organization from other modern or fossil scleractinian^14,19^. Because distinct patterns of TD organization are highly conservative traits in the evolution of scleractinian corals^63^, a unique micrabaciid fine-scale skeletal organization clearly suggests a long-period of independent evolutionary history in relation to gardineriids. On the other hand, although microstructural organization of Ordovician kilbuchophyllids is unknown (these fossils occur as moulds), these Paleozoic corals with scleractinian pattern of septal insertion had an epithecal wall (somewhat similar to gardineriids), and a pattern of bifurcations of higher septal cycles similar to micrabaciids. Together, these morphological characteristics allied to the molecular based phylogeny point towards a common and deep Paleozoic root for the order Scleractinia.

The early divergence of the azooxanthellate, solitary, deep-water micrabaciids and gardineriids (also supported by Stolarski et al.^14^ and Kitahara et al.^15^) contrasts with some hypotheses for whether first scleractinians were symbiotic and if they inhabited shallow or deep water environments^64^. In fact, Campoy and colleagues^16^ used four markers (18S rDNA, 28S rDNA, 16S rDNA and *cox1*) and 513 scleractinian coral species from almost all extant families and hypothesized that the first scleractinian would have been azooxanthellate and solitary. Nonetheless, symbiosis with zooxanthellae was widespread in Triassic corals^65^ and there is some degree of disagreement about it being lost and reappearing a few times^64,66^ or being gained only once during scleractinian evolution^16^. In contrast, it appears that coloniality was the first one to be gained and there is an agreement that it was lost and gained more than once^16,64^ and even the presence of multiple mouths in one polyp seems to be a labile trait in some families of this order as well (e.g. Dendrophylliidae^66^ and Fungiidae^67,68^).

Interestingly, all Paleozoic purported scleractiniamorph corals (i.e. *Kilbuchophyllia*, *Houchangocyathus,* and possibly *Numidiaphyllum*) were solitary polyps or had a loosely constructed phaceloid growth form (see Scrutton^69^; Ezaki^70–72^). Due to the several similarities with living solitary azooxanthellate scleractinians^69,72^, these aforementioned corals were purported to be azooxanthellate. Also, coral-zooxanthellae symbiosis has been established around 14 mya after the P/T boundary^65,73^, and although diagenetic conditions preclude unambiguous determination of a symbiotic or asymbiotic condition in Paleozoic corals, azooxanthellate and solitary lineages of living corals are the first to diverge in recent evolutionary reconstructions of the order Scleractinia. Such pattern is not limited to Micrabaciidae and Gardineriidae, but includes some lineages within “Complex” and “Robust” corals^11,14,15,74^. Examples are the families Anthemiphylliidae and Deltocyathidae in “Robust” and Fungiacyathidae, Turbinoliidae and Flabellidae in “Complex”^11,14–16,74^, showing that both clades present azooxanthellate deep-sea corals as first lineages to diverge. Hence, it would be more parsimonious to assume that the origin of the order is more likely azooxanthellate and solitary. Considering the coral fossil gap observed during the Lower Triassic, corals may have survived as azooxanthellate taxa living in the deep sea, as proposed by Ezaki^70^, and are too rare to be detected in the fossil record after the end-Permian extinction, as suggested by Stanley and Fautin^75^.

In terms of evolution inside Micrabaciidae, the recovered topology reinforces the observations made by Owens^76^ that *R. niphada* may be an intermediate species between *Rhombopsammia* and *Letepsammia* and, therefore, the first cycle septal solidity in the former and the total number of septa might not grant the split of both genera. Additional data from the remaining micrabaciid genera (i.e. *Leptopenus* and *Stephanophyllia*) will help to clarify such relationship and may shed light on deep-sea adaptations among scleractinian corals*.*

## Methods

Specimens belonging to four species of the family Micrabaciidae (*Letepsammia formosissima* (Moseley, 1876): IK-2012-3802; *L. franki* Owens, 1984: IK-2012-3748; *L. superstes* (Ortmann, 1888): IK-2012-3754; and *Rhombopsammia niphada* Owens, 1986: IK-2012-3832) were sourced from the *Muséum national d'Histoire naturelle* (Paris, France) Cnidaria collection. Total genomic DNA extraction was performed using the DNeasy Blood and Tissue kit (Qiagen) and libraries for Next Generation Sequencing (NGS) were prepared using TruSeq DNA Nano library preparation kit (Illumina; one library per species, based on one sample each). Since DNA from *R. niphada* was particularly degraded, Covaris shearing parameters were changed for this sample according to manufacturer suggestions (duty cycle: 5%; duration: 70 s). Moreover, in order to avoid adapter-dimer formation, adapters were diluted (3×) and the number of cycles at the PCR step was set to 12 cycles, following Illumina recommendations. Library concentrations were quantified on a Qubit 2.0 fluorometer and size distributions were assessed on a Bioanalyzer (Agilent). Samples were pooled with other libraries and sequenced on two different MiSeq v3 2 × 300 bp runs (*L. formosissima* in a run with other seven libraries and *L. superstes* and *R. niphada* in a different run with other eight). The same libraries from species *L. superstes* and *R. niphada* were also included on a NextSeq v2 High Output 2 × 75 bp pooled run with 16 samples in total. Illumina sequencing was performed at the Genome Investigation and Analysis Laboratory of the Centro de Facilidades para a Pesquisa, University of São Paulo. Raw sequences were trimmed using Trimmomatic^77^ under default settings, and the trimmed reads were used to assemble mitogenomes using MITObim^78^. Complete assembly of mitogenomes was ascertained by a circular mitogenome recovered, as determined by circules.py^78^. Assembled mitogenomes were then annotated by MITOS2^79^. Protein coding genes with start or stop codons that did not match the mold/coelenterate mitochondrial genetic code were inspected for the presence of suitable codons before the beginning or after the end of their annotations and re annotated accordingly. Data from previous attempts to sequence Micrabaciidae mitogenomes by primer walking (using the same samples; primer sequences and PCR settings from Lin et al.^74^) were used to refine *R. niphada* and *L. formosissima* assemblies. Data for *L. franki* were generated by Sanger sequencing followed by editing and assembling in Sequencher^80^.

A selection of published mitogenomes (Table 1) was downloaded from GenBank and re-annotated on MITOS2. Nucleotide sequences were aligned by gene (11 PCGss, 2 rRNAs and 2 tRNAS) or exon for multi-exon genes (i.e. *nad*5 and *cox1*) in MAFFT v7 using L-INS-i algorithm^81^. Alignments were tested for substitution saturation on DAMBE v7.0.12^82^. In saturated alignments, the third codon position nucleotide was removed and tested for saturation again. Only non-saturated alignments were concatenated using catsequences (https://github.com/ChrisCreevey/catsequences) and the final matrix is available at Zenodo (https://doi.org/10.5281/zenodo.4133805). For phylogenetic reconstructions, both maximum likelihood (ML) and Bayesian inference (BI) methods were used. The matrix was partitioned by either gene or exon in multi-exon genes. The former was carried out in RAxML v8.2.12^83^ with 1000 bootstrap replicates and 100 random starting trees (GTR + G model). The latter was performed in MrBayes v3.2.7^84^ on CIPRES portal^85^, after searching for the best substitution model for each alignment using Bayesian Information Criterion on jModelTest2^86^ (run on CIPRES portal). Two Markov chain Monte Carlo runs with four chains each were run for 20 million generations, sampling once every 1000 trees, and discarding the first 30% of them as burn-in, following run convergence check in Tracer v1.7.1^87^.

## Supplementary information

Supplementary Information.

## Data Availability

The data underlying this article are available in the GenBank Nucleotide Database at https://www.ncbi.nlm.nih.gov/nuccore/ and can be accessed with accessions MT705247, MT706034, MT706035 and MT706036–MT706040. The alignment used for phylogenetic reconstructions is available in Zenodo at https://doi.org/10.5281/zenodo.4133805.
