# Supplementary material for: The earliest diverging extant scleractinian corals recovered by mitochondrial genomes
Source: Sci Rep. 2020 Nov 26;10:20714. doi: 10.1038/s41598-020-77763-y (PMC7693180; doi:10.1038/s41598-020-77763-y)
Supplement: Supplementary file 1 — Supplementary Information. [file 41598_2020_77763_MOESM1_ESM.pdf]

The earliest diverging extant scleractinian corals recovered by mitochondrial genomes

Isabela G. L. Seiblitz[1,2]; Kátia C. C. Capel[2]; Jarosław Stolarski[3]; Zheng Bin Randolph Quek[4]; Danwei Huang[4,5]; Marcelo V. Kitahara[1,2]

[1]Departamento de Ciências do Mar, Universidade Federal de São Paulo, Santos, São Paulo, Brazil

[2]Centro de Biologia Marinha, Universidade de São Paulo, São Sebastião, São Paulo, Brazil

[3]Institute of Paleobiology, Polish Academy of Sciences, Warsaw, Poland

[4]Department of Biological Sciences, National University of Singapore, Singapore

[5]Tropical Marine Science Institute, National University of Singapore, Singapore

*Corresponding author: Isabela G. L. Seiblitz, isaseiblitz@gmail.com.

Supplementary Table S1: Number of reads obtained along the methodology i.e. from Illumina sequencing runs ("Raw"), after quality trimming ("Trimmed") and included in the Mitobim assembly ("Assembled").

	NextSeq			MiSeq		
**Species**	**Raw**	**Trimmed**	**Assembled**	**Raw**	**Trimmed**	**Assembled**
Letepsammia formosissima	N.A.	N.A.	N.A.	3,464,650	2,779,040	5,772
Letepsammia superstes	26,584,520	14,566,182	22,350	3,037,202	2,767,107	8,185
Rhombopsammia niphada	29,910,418	15,824,076	17,819	5,605,634	4,840,710	8,344
